# The Inflammatory Chemokine CCL5 and Cancer Progression

**DOI:** 10.1155/2014/292376

**Published:** 2014-01-02

**Authors:** Donatella Aldinucci, Alfonso Colombatti

**Affiliations:** ^1^Experimental Oncology 2, Aviano National Cancer Institute (CRO), Via Franco Gallini 2, 33081 Aviano, Italy; ^2^Department of Medical and Biological Science Technology, Microgravity Ageing Training Immobility Excellence Center (MATI), Piazzale M. Kolbe, 33100 Udine, Italy

## Abstract

Until recently, inflammatory chemokines were viewed mainly as indispensable “gate keepers” of immunity and inflammation. However, updated research indicates that cancer cells subvert the normal chemokine system and these molecules and their receptors become important constituents of the tumor microenvironment with very different ways to exert tumor-promoting roles. The CCR5 and the CCL5 ligand have been detected in some hematological malignancies, lymphomas, and a great number of solid tumors, but extensive studies on the role of the CCL5/CCR axis were performed only in a limited number of cancers. This review summarizes updated information on the role of CCL5 and its receptor CCR5 in cancer cell proliferation, metastasis, and the formation of an immunosuppressive microenvironment and highlights the development of newer therapeutic strategies aimed to inhibit the binding of CCL5 to CCR5, to inhibit CCL5 secretion, or to inhibit the interactions among tumor cells and the microenvironment leading to CCL5 secretion.

## 1. Introduction

Epidemiological and experimental studies provided clear evidence that unresolved pathogen infections and chronic inflammation promote tumor development and led to the inclusion of inflammation among the hallmarks of cancer [1, 2]. On the other hand, cancer cells not only make themselves “invisible” to the immune system, but also favor the formation of an immunosuppressive microenvironment unable to eliminate cancer cells [3]. As a result, the reduced secretion of molecules acting as tumor-promoting factors and the normalization of the tumor microenvironment [4] are main goals to develop appropriate antitumor strategies.

The tumor microenvironment is composed of stromal and inflammatory cells that are recruited and/or locally induced to proliferate or differentiate by tumor cells or by normal cells “educated” by tumor cells. They communicate directly through cell-cell contact but also indirectly through paracrine signals [4]. These signals are predominantly constituted by cytokines and chemokines (chemotactic cytokines), key orchestrators of leukocytes trafficking under homeostatic conditions as well as during inflammation and cancer [5] and part of the molecular pathways driving cancer cell survival, motility, and invasiveness [6].

Chemokines, identified on the basis of their ability to induce chemotaxis, have a fundamental role not only in inflammation and immune surveillance but also in cancer progression [7]. Chemokines, secreted by the tumor cells from primary tumors or metastatic sites or by the normal cells recruited and/or locally activated, can behave as growth factors [8], increase metastasis formation and angiogenesis, [9] or induce the formation of an immunosuppressive microenvironment. This last complex capacity is obtained by recruiting activating tumor-associated macrophages (TAM) [10], myeloid-derived suppressor cells (MDSC), T-regulatory cells (T-reg) [11], or mesenchymal stem cells (MSCs) [12] and by inhibiting the antitumor activity of Th1 cells and cytotoxic T lymphocytes (CTL) [13].

In response to chemokines present in remote organs, tumor cells that express the corresponding receptor disseminate with higher efficiency [14]. Furthermore, tumor cells acquire higher adhesive, migratory, and invasive properties in response to chemokines that are expressed at preferential metastatic sites [15]. As a consequence, the presence of inflammatory cells such as reactive leukocytes and the expression of a large number of inflammatory mediators (e.g., cytokines, chemokines, and enzymes) in the primary tumor are mostly associated with poor prognosis and metastasis formation [16].

A variety of chemokines and chemokine receptors have been detected in neoplastic tissues [1, 4]. We will focus our attention primarily on the C-C chemokine ligand 5 (CCL5), also known as Regulated upon Activation, Normal T-cell Expressed, and Secreted (RANTES), and its receptors C-C chemokine receptor type 5 (CCR5).

CCR5 is a seven-transmembrane G-protein-coupled receptor, mediating diverse signaling cascades in response to its ligands. CCR5, a promiscuous receptor, binds with high-affinity CCL5, CCL3 (MIP-1a), and CCL4 (MIP-1b) and is the major coreceptor for HIV [17].

CCL5 belongs to the C-C chemokine family whose members also include CCL3 and CCL4 [18]. CCL5, a target gene of NF-*κ*B activity, is expressed by T lymphocytes, macrophages, platelets, synovial fibroblasts, tubular epithelium, and certain types of tumor cells [18]. NF-*κ*B activation by different stimuli such as CD40L [19] or IL-15 [20] induces CCL5 production.

CCL5 plays an active role in recruiting a variety of leukocytes into inflammatory sites including T cells, macrophages, eosinophils, and basophils. In collaboration with certain cytokines that are released by T cells such as IL-2 and IFN-*γ*, CCL5 also induces the activation and proliferation of particular natural killer cells to generate C-C chemokine-activated killer cells [18]. CCL5 produced by CD8^+^ T cells and other immune cells has been shown to inhibit HIV entry into target cells.

CCL5 activity is mediated through its binding to CCR1, CCR3, and mainly CCR5 [18]. CCR4 [21, 22] and CD44 are auxiliary receptors for CCL5 [21, 23].

The exact functions of CCL5 in tumor biology are still unclear. CCL5 production is relevant to inducing proper immune responses against tumors [2], but, on the other hand, CCL5 is associated with cancer progression and metastasis.

CCL5/CCR5 interactions may favor tumor development in multiple ways: acting as growth factors, stimulating angiogenesis, modulating the extracellular matrix, inducing the recruitment of additional stromal and inflammatory cells, and taking part in immune evasion mechanisms [3].

**Figure 1:**
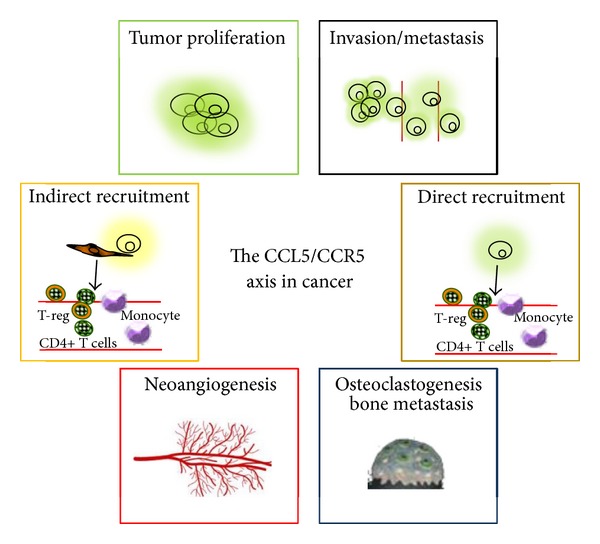
Effects of the CCL5/CCR5 interactions in cancer. Cancer cells secrete CCL5 or induce fibroblasts to secrete CCL5 which act in a paracrine or autocrine fashion on CCR5-positive tumor cells to sustain their proliferation, to recruit immunosuppressive cells (T-reg cells, monocyte), to induce osteoclasts activation and bone metastasis, to induce neoangiogenesis, and to guide tumor cells to disseminate to distant organs.

A schematic view of the consequences of the CCL5/CCR5 interactions in cancer is shown in Figure 1.

This review summarizes updated information on the role of the CCL5/CCR5 axis in tumor development and/or progression, focusing primarily on multiple myeloma (MM), classical Hodgkin lymphoma (cHL), prostate, breast, gastric, colon, and ovarian cancer, and melanoma.

Based on the findings obtained so far, we propose that inflammatory chemokines and their receptors are attractive therapeutic targets in malignancy.

## 2. Mechanism Leading to Cancer Cell Proliferation and Metastasis Formation via the CCL5/CCR5 Axis 

The binding of chemokine to their G-protein-coupled receptors (GPCRs) activates a series of downstream effectors that facilitate internalization of the receptor and signal transduction leading to integrin activation (adhesion) and polarization of the actin cytoskeleton [24]. The consequences are directional sensing, cell polarization, accumulation of small GTPases, Rac, Cdc42, and PI3K at the leading edge, actin polymerization, and F-actin formation. These changes cause actomyosin contraction and tail retraction and finally cell migration [24].

More specifically, CCL5 contributes to the activation of the *α*v*β*3 integrin and to cell migration through PI3K/Akt, which in turn activates IKKalpha/beta and NF-*κ*B [25]. NF-*κ*B activation also can elevate the secretion of MMP-9 [26] or promote invasion by increasing the secretion of both MMP-2 and -9 and by activating the ERK and Rac signaling [27]. CCL5 induces migration also by upregulating the activities of MMP-9 through STAT3 [28]. In other instances CCL5/CCR5 acts via MEK, ERK, and then NF-*κ*B, resulting in the activations of *α*v*β*3 integrin and contributing to cell migration [29].

Chemokines, by activating the tyrosine kinase receptors, the Jak-STAT, or the MAPK/ERK signaling pathway, also promote tumor cell proliferation [30]. Exogenous CCL5 stimulates cell proliferation by inducing the mTOR pathway, leading to a rapid upregulation of cyclin D1, c-Myc, and Dad-1 expression. An additional mechanism based on the CCL5-CCR5 interaction can lead to increased cell proliferation: increased glucose uptake, increased ATP production, and enhanced glycolysis, associated with extracellular acidification [31].

## 3. The CCL5/CCR5 Axis in Hematological Malignancies

Many studies were published during the last several years on the expression of CCL5 and CCR5 in hematological malignancies, but, only for multiple myeloma (MM) and at least in part for cHL, we have a comprehensive view of the role played by the CCL3-CCL5/CCR5 pair.

### 3.1. Multiple Myeloma

The MM cell localization in the bone marrow and the cross-talk with the bone niche trigger dramatic alterations in the bone marrow (BM) microenvironment, critical for tumor progression, resistance to therapies, and osteolytic bone destruction [32]. The interaction between osteoclasts (OCs) and MM cells plays a key role in the pathogenesis of MM-related osteolytic bone disease. MM cells promote OCs formation and, in turn, OCs enhance tumor cell proliferation via cell-cell contact [33].

The CCR5-ligand CCL3 is detected in MM cell line and freshly isolated MM cells [34, 35] and is one of the most important OC-activating factors produced by MM cells and a contributor of MM-associated osteolytic bone disease [36]. MM cells from patients with multiple bone lesions secrete higher amounts of CCL3 (and CCL4) than those with less-advanced bone involvement [37]. Consistently, CCL3 serum levels are elevated in newly diagnosed MM patients and correlate with the extent of bone disease, bone resorption, and disease prognosis [38]. Increased expression of CCL3 in bone biopsies correlates with extensive bone disease, increased angiogenesis, and advanced stage in newly diagnosed patients with MM [39]. CCL3, secreted by MM cells, stimulates OC activity and also inhibits osteoblast formation, further contributing to the imbalance between bone resorption and bone formation [40]. MM cells also secrete CCL5, suggesting a possible role of this chemokine in the pathogenesis of MM since, like CCL3, it is a potent activator of both CCR1 and CCR5 receptors [41] expressed by stromal cells and OC precursors [35].

Several studies have evaluated the expression of CCR5 and CCR1 by MM cell lines and by cells derived from patients [34, 35, 42–45] and demonstrated that their engagement determines MM cell survival, migration, and homing to the BM. In fact, MM cells migrate in the presence of CCL5 and the extent of migration depends on the CCR5 expression levels [35, 43, 45].

Inhibition of CCR1 and CCR5 receptors by antagonists or neutralizing antibodies partially reduce osteoclastogenesis, osteolytic lesions, and MM-induced angiogenesis [34, 35, 42]. Recently, Dairaghi et al. [46] demonstrated that CCR1 blockade by the selective antagonist CCX721 reduces tumor burden and osteolysis in vivo in a mouse model of myeloma bone disease, likely by inhibiting the cross-talk of MM cells with OCs and OC precursors [46]. Thus the development of CCR1 antagonists for the treatment of MM and associated osteolytic bone disease is a further therapeutic possibility.

Overall, the current observations propose two major mechanisms by which CCL3 and/or CCL5 released by tumor cells and their receptors support MM progression: the first is the ability to disrupt bone homeostasis and induce bone destruction, and the second is the bone marrow homing of MM cells [35] due to the expression of CCR5 and CCR1. Therefore, counteracting the consequences of these chemokine/chemokine receptors interactions may represent a new therapeutic option in MM.

### 3.2. Hodgkin Lymphoma

The microenvironment is essential for growth and survival of classical Hodgkin Lymphoma (HL) tumor cells [8] and chemokines play a primary role in its formation. They may exert a direct action on tumor cells by increasing cell survival and proliferation, recruit cells capable of sustaining the growth of tumor cells by providing a suppressive environment that suppresses cytotoxic immune responses, or redirect HL cells to advantageous microenvironmental sites within the lymphoid tissues.

cHL cells secrete cytokines/chemokines and express a variety of cytokine/chemokine receptors [8, 47] and it is now widely assumed that the clinical and histological features of cHL are primarily due to the effects of a plethora of cytokines and chemokines secreted by cHL cells such as CCL5 [48, 49], CCL17 [47], CCL22 [50], CCL28 [51], and CCL20 [39, 52] or by the surrounding cellular infiltrate. The recruitment and proliferation of nontumor cells may be also mediated by molecules produced by “normal” cells of the microenvironment, activated by tumor cells [8]. For example: cHL cells (i) do not express eotaxin but produce IL-13 and TNF-*α* which are capable of inducing eotaxin expression in cocultured dermal fibroblasts in a concentration leading to a specific chemotactic response of Th2 cells [53]; (ii) produce molecules capable of inducing CCL5 secretion in HL-derived fibroblasts [49]; (iii) express CD40 and its engagement by CD40L rosetting T-cells increase CCL5 secretion [49]. Together these lines of evidence suggest that the cross-talk between tumor cells and fibroblasts or the activation by surrounding CD40L+ T-cells may be involved in the influx and further proliferation of inflammatory cells typical of the HL microenvironment. Accordingly, when compared with control lymph nodes or tissues diagnosed with reactive lymphoid hyperplasia, cHL tissues display higher levels of chemokines such as CCL5 and CCL3 [47, 48]. Both chemokines are significantly higher in EBV-positive than in EBV-negative HL tissues [47], consistent with the fact that the EBV gene LMP1 is necessary to induce the expression of CCL5 in EBV-negative cell lines [54].

Both CCR5 [49] and CCL5 [48, 49] are also constitutively expressed by cHL-derived cell lines (L-428, KM-H2, L-1236, and L-540), by tumor cells from cHL lymph node tissues, and by bystander cells including lymphocytes and macrophages [49]. CCR5 receptor is functional since human recombinant CCL5 increases the clonogenic growth of cHL tumor cells. As a consequence, CCL5 secreted by the microenvironment may be a paracrine growth factor for cHL cell. Consistent with the fact that cHL cell lines expressed the CCR5 receptor and its ligand, neutralizing anti-CCL5 mAbs decrease the spontaneous clonogenic growth, suggesting that CCL5 may represent an autocrine growth factor for cHL cells. CD40 engagement [19] and cocultivation with fibroblasts from HL-involved lymph nodes (HLF) [49] increase CCL5. On the other hand, the silencing of IRF4, a transcription factor iperexpressed by cHL cells and whose expression is linked to proliferation and survival [55], decreases CCL5 secretion. CCL5 secreted by cHL cells increases the migration of mast cells [48], eosinophils, CD4+ T cells [49], and likely T-reg cells [56], highlighting its involvement in the formation of the microenvironment [8].

**Figure 2:**
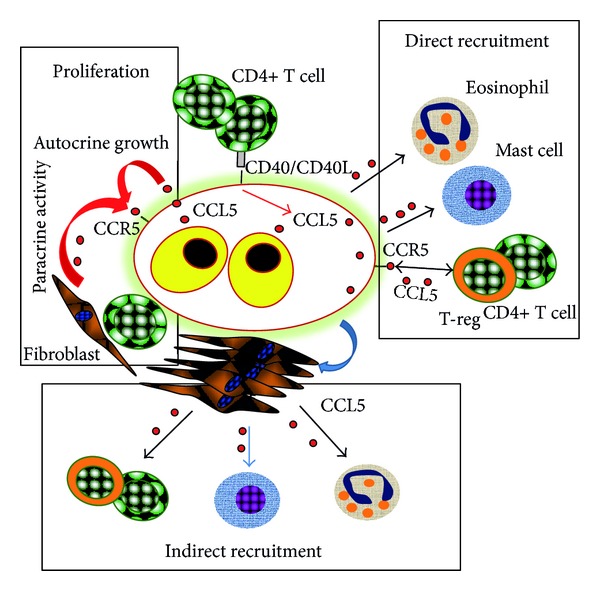
Interactions among cHL cells and the microenvironment. Proposed role of the CCL5/CCR5 axis in Hodgkin Lymphoma leading to tumor cell proliferation and microenvironment formation. CCL5 produced by cHL cells may represent an autocrine growth factor. CCL5 secreted by T cells or fibroblasts may represent a paracrine growth factor. CD40L increases CCL5 secretion by cHL cells and they induce fibroblasts to secrete CCL5. CCL5 secreted by cHL (direct recruitment) or fibroblasts activated by HL cells (indirect recruitment) may in turn recruit CD4+ T cells, T-reg cells, eosinophils, and mast cells.

As a consequence CCL5 and the CCR5 ligands secreted by tumor cells or by the surrounding T-cells, macrophages, or fibroblasts may support cHL progression by increasing proliferation and by recruiting cells involved in the microenvironment formation. A schematic view of the possible mechanisms (paracrine and autocrine) leading to cHL cells proliferation and microenvironment formation by CCL5 is shown in Figure 2.

## 4. The CCL5/CCR5 Axis in Solid Tumors

A number of solid tumor types express CCL5 and/or CCR5, but only some malignancies were widely studied, thus providing evidence of the involvement of this pair in cancer progression and development. We briefly summarize the role of CCL5/CCR5 in melanoma and gastric, ovarian, cervical, colorectal, and prostate cancer. However, since the most extensive results were obtained in breast cancer, major emphasis is given to this malignancy.

### 4.1. Breast Cancer

CCL5, while being minimally expressed by normal breast epithelial duct cells, is highly expressed by breast tumor cells at primary tumor sites, regional lymph nodes, and metastatic sites, indicating that CCL5 expression is acquired in the course of malignant transformation [18] and that CCL5 plays a role in breast cancer development and/or progression. Increased positivity and expression levels of CCL5 by breast tumor cells are significantly associated with [57] disease progression, relapse, and/or metastasis, compared to patients in remission [58, 59]. In this tumor the major source of CCL5 is the tumor cells [57]; however, CCL5 is also expressed by infiltrating leukocytes and mesenchymal stem cells (MSCs) of the tumor microenvironment [15, 57, 60]. CCL5 is also present in interstitial fluids perfusing the tumor, in pleural effusions, and in serum [18].

A functional CCR5 receptor is expressed by a subpopulation of human breast cancer cell lines and displays a functional response to CCL5. In addition, oncogene transformation induces CCR5 expression, and the subpopulation of cells that express a functional CCR5 also displays increased cell migration [61] and invasiveness [62]. A microarray analysis on 2,254 human breast cancer specimens found increased expression of CCL5 and its receptor CCR5, but not CCR3, in the basal and HER-2 genetic subtypes [62]. In contrast, when a similar analysis was performed in nonneoplastic breast samples, no correlation between CCL5 and CCR5 expression levels was found, indicating that CCL5/CCR5 signaling may be preferentially activated during the development of specific breast cancer subtypes [62]. CCL5 expression is strongly associated with the progression of breast cancer, particularly the triple-negative breast cancer (TNBC), and may represent an immunotherapeutic target in the TNBC [63].

Hypoxia is a major selective factor that promotes the growth of tumors with a diminished susceptibility to radiation and chemotherapy and is associated with cancer progression, cancer metastasis, and thus poor prognosis. Hypoxia induces a strong increase of both CCL5 and CCR5 expressions by breast cancer cells [64]. Under this experimental condition CCL5 stimulates cell migration rather than cell proliferation and neutralization of CCL5 inhibits the hypoxia-induced migration of cancer cells. Similarly, overexpression of CCR5 increases cell migration, and knockdown of CCR5 attenuates hypoxia-mediated cell migration. Hypoxia-inducible factor-1*α* (HIF-1*α*) is involved in CCR5 and CCL5 regulation under hypoxia and HIF-1*α* mRNA levels are highly correlated with CCR5 mRNA and CCL5 mRNA levels in clinical samples [64].

CCL5 also concurs with the cross-talk between breast cancer cells and MSCs: cancer cells stimulate CCL5 secretion by MSCs and osteoblasts of the tumor microenvironment and CCL5 in turn induces tumor cell migration and promotes invasion and metastasis [15, 60]. MSCs-derived CCL5 promotes mammary tumor cell invasion and the activation of matrix metalloproteinases (MMPs), consistent with the fact that CCL5 is capable of upregulating the release of MMP-9 [65].

Tumor infiltrating cells seem bona fide prognostic and even predictive biomarkers and could be incorporated into diagnostic and therapeutic algorithms of breast cancer [18]. CCL5 supports breast malignancy by changing the equilibrium between leukocyte infiltrates in tumors, leading to dominance of cells with tumor-promoting rather than tumor-killing activities. In fact, CCL5 shifts the balance between different leukocyte cell types by increasing the presence of deleterious TAMs [10] that secrete proangiogenic factors, suppress the antitumor response [66], and inhibit the antitumor T-cell activities.

CCL5, together with tumor-derived colony-stimulating factors, promotes mammary tumor progression generating MDSCs in the bone marrow, helping to maintain the immunosuppressive capacity of human MDSCs [67]. CCL5 neutralization could decrease the immunosuppression activity of MDSCs, improve the efficacy against poorly immunogenic tumors, and reduce progression and metastasis.

CCL5 expression by breast tumor cells represents a valuable prognostic factor for detection of stage II breast cancer patients who are at risk for disease progression [68]. Its expression is associated with the absence of estrogen receptor, thus increasing the prognostic value of each of these two markers in patients (in the order III > II > I) at risk for progression [68]. CCL5 serum levels are elevated in breast cancer patients compared to healthy individuals [69] and tend to be higher in lymph-node-positive patients, larger tumor size, the presence of lymphovascular invasion and multifocal tumors [70].

CCL5 is also involved in drug resistance [71]. Tamoxifen resistance is a major therapeutic problem in breast cancer and a significant correlation between STAT3-RANTES autocrine signaling and acquisition of tamoxifen resistance has been reported: STAT3 and RANTES in tamoxifen-resistant MCF-7 cells regulate each other via autocrine signaling, leading to the induction of an antiapoptotic signal. This latter facilitates the maintenance of drug resistance, thus suggesting a novel strategy for the management of patients with tamoxifen-resistant tumors [71].

To conclude, based on several studies done in patients, animal model systems, and in vitro systems, the CCL5/CCR5 axis seems to have a crucial role in cancer progression and may represent an important breast cancer therapeutic target with minimal adverse impact [63].

### 4.2. Melanoma

Melanoma cell lines and melanoma tissues express a number of chemokines that support their growth and are implicated in tumor progression [72]. Furthermore, organ-specific patterns of melanoma metastasis correlate with the expression of specific chemokine receptors [72].

CCL5 and CCR5 are expressed by melanoma cells, primary melanomas, and cutaneous metastasis. CCL5 is higher in melanoma cells than in normal melanocytes and is associated with a higher malignancy state and increased tumor formation [73, 74]. CCR5 is exclusively expressed in primary melanomas and some cutaneous metastases [75]. Recently, to better evaluate the significance of CCR5 expression in melanoma development, tumor growth in CCR5 knockout (CCR5^−/−^) and wild type (CCR5^+/+^) mice was investigated. CCR5 deficiency caused apoptotic melanoma cell death through inhibition of NF-*κ*B and upregulation of IL-1R*α* [76], thus suggesting a tumor-promoting role of CCR5. Already a previous study by Mellado et al. had shown that CCR5 plays a key role in inducing apoptotic death in tumor infiltrated lymphocytes (TIL) in a CCL5-dependent manner: CXCL12 released by melanoma cells induced the expression of CCL5 by TIL, which in turn activated their death program [77]. This activity is upregulated also by CCL3 and CCL4: they act via CCR5 to induce cytochrome-c release into the cytosol, leading to activation of caspase-9 and -3. Recently, using a mouse model of melanoma, Schlecker et al. [13] demonstrated that tumor-infiltrating monocyte-MDSCs directly attract high numbers of T-regs via CCR5 and that intratumoral injection of CCL4 or CCL5 increases tumor-infiltrating T-regs, but CCR5 deficiency led to their profound decrease. Moreover, melanoma growth is delayed in CCR5-deficient mice, likely because of a profound decrease of T-regs, emphasizing the importance of CCR5 in the control of antitumor immune responses.

The conclusion is that the CCL5/CCR5 axis seems associated with melanoma progression due to increased levels of immunosuppressive cells.

### 4.3. Gastric Cancer

Increased CCL5 levels are expressed by human gastric cancer cell lines characterized by a high metastatic potential [78] suggesting a tumor-promoting role of CCL5 in gastric cancer. This possibility is supported by the effects of supernatants derived from low- and high-metastatic gastric cancer cell lines on the activities of peripheral blood mononuclear cells (PBMC). Supernatants from high-metastatic gastric cancer cell lines increase CCL5 expression in PBMC, as compared to PBMC stimulated by supernatants of low-metastatic cells. In turn, tumor cells cocultured with PBMC have higher invasion properties than noncocultured cells, and this process is highly inhibited by antibodies to CCL5 [79], suggesting that the cross-talk with PBMC, likely through CCL5, increases the invasion potential of tumor cells.

Several authors have then analyzed CCL5 expression in gastric cancer and found a possible correlation with the formation of metastasis. The possibility that CCL5 could serve as a predictor of metastasis was based on a study analyzing CCL5 circulating levels prior to anticancer treatment: CCL5 levels are higher in patients than in healthy controls; furthermore, the expression is higher in stage IV patients than in stages I or II-III [80] and in metastatic sites [81]. In fact, CCL5 and CCR5 are highly expressed in gastric cancer with lymph node metastasis, and CCL5 levels in the lymph nodes with cancer invasion are substantially increased, confirming the role of CCL5/CCR5 axis in metastasis formation [81].

Following infection with *Helicobacter pylori* in a gastric cancer model system CCL5 is elevated and its levels are reduced by treatment with anti-inflammatory drugs [82]. This is in accordance with the finding that IL-2 and IFN-*γ* (Th1 cells) are lower and IL-10 (Th2 cells) is higher in lymph node metastasis than in cancer without metastasis, suggesting a shift toward an immunosuppressive microenvironment [82].

Expression of CCR5 by gastric tumor cells is associated with a lower survival rate [83]. Gastric cancer cells exploit CCL5, not only for their own growth, but also to assist in evasion of the host immune system [84]. CCL5 serum levels correlate with the clinical stage and treatment with CCL5 promotes tumor growth. Gastric cancer cells stimulate CD4+ T lymphocytes to secrete CCL5 and they may also induce Fas-FasL-mediated apoptosis of CD8+ T lymphocytes using CCL5 [84].

The conclusion is that the CCL5/CCR5 axis seems associated with gastric cancer progression due to increased growth and metastasis formation.

### 4.4. Colon Cancer

The CCL5/CCR5 axis plays also a role in colon cancer since CCL5 and its receptors are overexpressed within primary as well as liver and pulmonary metastases compared to healthy tissues [85]. CCL5 increases the in vitro growth and the migratory responses of colon cancer cells from both human and mouse origins. In addition, systemic treatment of mice with neutralizing anti-CCL5 antibodies reduced the extent of subcutaneous tumors, liver metastases, and peritoneal carcinosis. More recently, a novel mechanism of immune escape mediated by CCL5 was defined by Chang et al. [11]. Knockdown of CCL5 from CT26 mouse colon tumor cells decreases apoptosis of tumor-infiltrating CD8+ T cells and reduces tumor growth in mice. Here, CCL5 not only promotes migration of T-reg cells to tumors but also enhances the killing ability on CD8+ T cells. This augmented function is associated with the increased release of TGF-*β* by T-reg cells [11].

While a treatment with TAK-779, a CCR5 antagonist, only partially compromises colon progression, CCL5 neutralization renders the tumors more sensitive to a PDGFR*β*-directed strategy in mice. It is of note that this combination regimen offers the greatest protection against liver metastases and fully suppresses macroscopic peritoneal carcinosis. The conclusion is that CCL5/CCR5 signaling recruits T-regs which in turn eliminate CD8+ T cells, thereby defining a novel mechanism of immune escape in colorectal cancer and pointing to the potential value of CCL5 as a therapeutic target [11].

### 4.5. Prostate Cancer

The CCL5/CCR5 axis is involved also in prostate cancer (PCa) progression: both are expressed in human prostate cancer (PCa) cell lines, primary cultures of prostatic adenocarcinoma cells, and PCa tissues [86]. CCL5 stimulates PCa cell proliferation and invasion and both are inhibited by the CCR5 antagonist TAK-779 [87]. CCL5 increases PCa proliferation in synergy with IL-6 and it is also induced by the antibody-mediated aggregation of the prostate specific membrane antigen (PSMA) [88]. PSMA is a type-II integral membrane protein capable of activating the NF-*κ*B transcription factor [88], predominantly localized to the epithelial cells of the prostate gland and whose expression increases several fold in high-grade prostate cancers and in metastatic and in androgen-insensitive prostate carcinoma [88].

Serum CCL5 levels do not differ among prostate cancer patients with or without paclitaxel resistance but the expression of the CCR1 receptor increases in paclitaxel-resistant PC3 prostate cancer cells [27]. Interaction between CCR1 and CCL5 promotes the invasion of taxane-resistant PC3 prostate cancer cells by increasing the secretion of MMP-2 and -9 via ERK and Rac activation [27] suggesting that CCR1 could be a novel therapeutic target for taxane-resistant prostate cancer.

### 4.6. Ovarian Cancer

CCL5 expression is detected not only in malignant ovarian biopsies, but also in normal biopsies, with minimal expression in ovarian cancer cell lines [89]. The cell types expressing CCL5 in the biopsies are not yet determined, but it is likely that infiltrating leukocytes constitute the major origin of this chemokine in ovarian tumors [89].

However, recently Long et al. [26] demonstrated that CCL5 is expressed in ovarian cancer stem cells (CSLCs) characterized by the expression of CD133 antigen that identifies a specific subpopulation of human ovarian cancer cell line and ovarian cancer tissue in which migration and invasion are particularly enhanced. In comparison to CD133-negative non-CSLCs, CCL5 and its receptors, CCR1, CCR3, and CCR5, are consistently upregulated in CD133-positive cells, and blocking of CCL5, CCR1, or CCR3 effectively inhibits the invasive capacity of CSLCs. The enhanced invasiveness is mediated through NF-*κ*B activation along with elevated MMP-9 secretion, suggesting that the autocrine activation of CCR1 and CCR3 by CCL5 represents one of the major mechanisms underlying the metastatic property of ovarian cancer cells [26].

Evidence supporting an association between CCL5 and ovarian carcinoma progression was also provided by a study analyzing chemokine levels in plasma of patients at different stages of disease. CCL5 levels are higher in ovarian cancer patients than in patients diagnosed with benign ovarian cysts and elevated in stages III-IV of ovarian cancer compared to stages I-II [90]. CCL5, along with CCL3 and CCL4, is present in ascitic fluids of ovarian carcinoma patients, and their levels positively correlate with the extent of T lymphocytes infiltration [91]. CCR5 and CCR1 are mainly detected in T lymphocytes and monocytes but only low expression of CCR5 is detected in the tumor cells [26, 91].

Cancer-associated fibroblasts (CAFs) are fibroblasts altered by the continuous exposure to cancer cells residing within the tumor microenvironment [92]. CAFs promote cancer cell invasion, proliferation, and metastasis by secreting cytokines and chemokines, which stimulate receptor tyrosine kinase signaling and epithelial-mesenchymal transition (EMT) programs [2].

The cultivation of ovarian cancer cells with normal fibroblasts generates CAFs possibly through the secretion of molecules that regulate the expression of miRNAs, noncoding RNA molecules that regulate gene expression at a posttranscriptional level [93]. The cross-talk between ovarian cancer cells and fibroblasts decreases *miR-31* and *miR-214* and increases *miR-155* expression, reprogramming normal fibroblasts into tumor-promoting cancer-associated fibroblasts. CCL5 is a key target of *miR-214* and the downregulation of *miR-214* increases CCL5 production, leading to increased tumor growth [94]. Anti-CCL5 antibodies block the effect of CAFs on tumor growth and cell migration [94] and CCL5-transfected normal fibroblasts increase the invasion of ovarian cancer cells [94], suggesting that CCL5 is a candidate effector molecule in CAFs, contributing to tumor cell recruitment and growth. The conclusion is that CCL5 is a protumorigenic chemokine and a key target of *miR-214*, thus showing that microRNA perturbation in the stromal microenvironment can affect tumor growth by increasing the secretion of CCL5 by CAFs and suggesting that CCL5 is a possible therapeutic target in ovarian cancer.

However, at present several outstanding questions remain and the roles played by CCL5 and its receptors in ovarian cancer are far from being resolved. Many additional aspects should be studied in this disease since they may provide important considerations and new strategies for therapeutic intervention.

## 5. Possible Clinical Applications: CCL5 and CCR5 as Therapeutic Targets in Cancer

A fundamental objective in cancer therapy is to disrupt the interactions leading to tumor growth or to the formation of a protumorigenic and immunosuppressive microenvironment. Accordingly, our knowledge on the role of chemokine receptors in proliferation and invasion of malignant cells and the role of chemokines in the recruitment of tumor-promoting myeloid cells or lymphocytes could be exploited in new approaches to treatment.

### 5.1. Inhibition of CCR5/CCL5 Interactions

CCR5 is an essential coreceptor for HIV virus entry to host cells and has therefore become an attractive target for anti-HIV therapeutics development. A number of specific small molecule CCR5 antagonists that are being used as antiviral therapies, but are also effective in blocking CCR5 signal transduction, were identified by high-throughput screening efforts. Maraviroc and vicriviroc are CCR5 antagonists that exert potent blocking activities for chemokine function and HIV entry [87]. There are several lines of evidence suggesting possible clinical applications of CCR5 antagonists in cancer treatment. Maraviroc or vicriviroc reduces in vitro invasion of basal breast cancer cells without affecting cell proliferation or viability [95, 96]. Maraviroc, that has already been licensed by FDA for the use in humans, prevents the development of hepatocellular carcinoma [97] in a mouse model and decreases pulmonary metastasis in a preclinical mouse model of breast cancer [62, 96], suggesting that CCR5 antagonists could be used as an adjuvant therapy to reduce the risk of metastasis in patients with the basal breast cancer subtype.

The nonpeptide CCR5 antagonist TAK-779 is a small molecular weight quaternary ammonium derivative, that binds exclusively to CCR5. It inhibits HIV infection [87] but also the CCL5-induced proliferation and invasion of PCa cells; this suggests that this antagonist may potentially be an effective inhibitor 9 of tumor growth and progression [86].

Anibamine [98, 99] is the first natural product reported as a CCR5 antagonist and thus provides a novel structural skeleton distinct from other lead compounds that have generally been identified from high-throughput screening efforts. Anibamine produces significant inhibition of PCa and ovarian cancer cell line OVCAR-3 proliferation without any significant cytotoxicity in NIH 3T3 fibroblastic cells [99, 100], suppresses adhesion and invasion of the highly metastatic M12 PCa cell line, and decreases PCa growth in mice [98, 99]. Based on these results, anibamine and also one of its synthetic analogues are potential leads to develop novel agents against prostate and ovarian cancer. Anibamine is currently undergoing further preclinical characterization [99, 100].

### 5.2. Inhibition of CCL5 Secretion

Inhibition of CCL5 secretion by cancer cell or by the tumor microenvironment may represent an additional system to affect cancer progression. MSCs are recruited by developing breast tumors where they can enhance the metastatic potential of weakly tumorigenic breast cancer cells through the secretion of CCL5 [15]. Zoledronic acid significantly affects the secretion of CCL5 and interleukin 6 in MSCs [101] suggesting that the drug could contribute to antitumor activity by affecting the ability of MSCs to interact with breast cancer cells. Alternatively, chemotherapy drugs could affect both proliferation and the formation of an immunosuppressive microenvironment by decreasing the secretion of CCL5 by cancer cells, as reported for the PI3K*δ*-specific inhibitor GS-1101 in cHL cells [9].

### 5.3. Inhibition of Cross-Talk (CCL5 Secretion)

Another therapeutical modality that deserves some consideration deals with the potential utilization of the cross-talk between cancer cells and cellular constituent of the microenvironment. Along this line we recently found that the EGFR-tyrosine kinase inhibitor gefitinib negatively affects EGFR activation by PC3-CM leading to decreased secretion of CCL5 by MSCs [102].

Overall, anti-CCL5 drugs could affect both tumor cell proliferation and/or the formation of an immunosuppressive microenvironment by decreasing the secretion of CCL5 by cancer cells.

**Figure 3:**
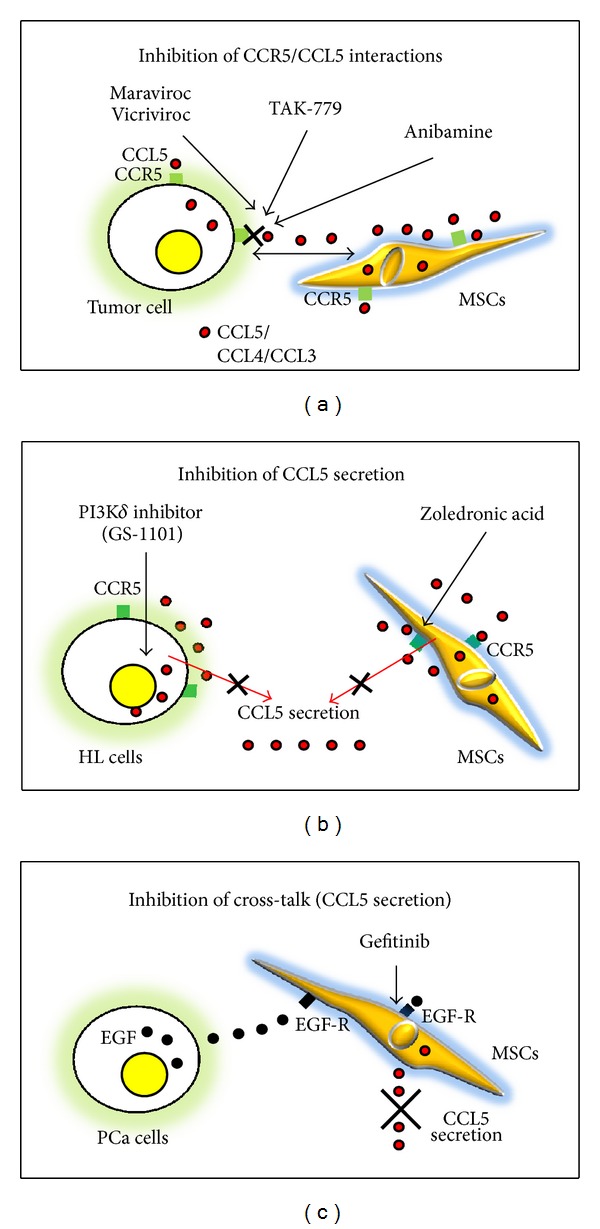
CCL5 and CCR5 as therapeutic targets in cancer. Different strategies proposed to disrupt the CCL5/CCR5 axis. (a) CCL5/CCL5 interaction may be inhibited by CCR5 antagonists. (b) CCL5 secretion by tumor cells or by MSCs of the tumor microenvironment may be decreased by treatment with chemotherapeutic agents. (c) The interactions between tumor cells and MSCs, mediated by EGF/EGFR pair, leading to increased CCL5 secretion by MSCs, may be inhibited by the EGFR-tyrosine kinase inhibitor gefitinib.

A schematic view of the possible therapeutic application is shown in Figure 3.

## 6. Conclusions

The investigation of the roles played by CCL5/CCR5 in tumor development and metastasis is only in its infancy. While promalignancy effects are strongly implicated in MM and breast cancer, their contribution to other malignancies such as cHL, melanoma, gastric, prostate, and ovarian and colon cancer deserves further studies.

Furthermore, one has to take into account the fact that the CCL5/CCR5 axis acts in conjunction with other chemokines to affect the malignant phenotype (e.g., the CXCL12/CXCR4 pair), exemplifying the multifactorial nature of malignancies and the need to target several mediators simultaneously. Also, in considering CCL5/CCR5 as therapeutic targets, we should evaluate the effects of anti-CCL5/CCR5 treatments on the immune integrity of the host. The optimal therapeutic modalities would have to accommodate two opposing demands: the need to inhibit the detrimental involvement of CCL5 and CCR5 in specific malignant diseases protecting their potentially beneficial activities in immunity, including the anticancer immune responses.

Overall, our current knowledge leads us to suggest the CCL5/CCR5 axis as a potential therapeutic target in several cancer diseases. However, bringing this proposal into practical application requires further research to more clearly elucidate the effects of CCL5 on cancer progression and the formation of an immunosuppressive microenvironment to insure that such treatments are supported by the appropriate rationale.

Finally, as postulated by Schall and Proudfoot, [103] the right target selection, time of intervention, and, in particular, functional dose may be the key to developing successful chemokine-targeted drugs not only for inflammatory diseases but also for cancer.
